# Pleural mesothelioma and lung cancer risks in relation to occupational history and asbestos lung burden

**DOI:** 10.1136/oemed-2015-103074

**Published:** 2015-12-29

**Authors:** Clare Gilham, Christine Rake, Garry Burdett, Andrew G Nicholson, Leslie Davison, Angelo Franchini, James Carpenter, John Hodgson, Andrew Darnton, Julian Peto

**Affiliations:** 1 London School of Hygiene and Tropical Medicine, London, UK; 2 Health and Safety Laboratory, Buxton, UK; 3 Department of Histopathology, Royal Brompton and Harefield Hospitals NHS Foundation Trust, and National Heart and Lung Institute, Imperial College, London, UK; 4 Department of Cellular Pathology, Leeds Teaching Hospitals NHS Trust, Leeds, UK; 5 Medical Research Council Clinical Trials Unit, Kingsway, London, UK; 6 Health and Safety Executive, Bootle, UK

## Abstract

**Background:**

We have conducted a population-based study of pleural mesothelioma patients with occupational histories and measured asbestos lung burdens in occupationally exposed workers and in the general population. The relationship between lung burden and risk, particularly at environmental exposure levels, will enable future mesothelioma rates in people born after 1965 who never installed asbestos to be predicted from their asbestos lung burdens.

**Methods:**

Following personal interview asbestos fibres longer than 5 µm were counted by transmission electron microscopy in lung samples obtained from 133 patients with mesothelioma and 262 patients with lung cancer. ORs for mesothelioma were converted to lifetime risks.

**Results:**

Lifetime mesothelioma risk is approximately 0.02% per 1000 amphibole fibres per gram of dry lung tissue over a more than 100-fold range, from 1 to 4 in the most heavily exposed building workers to less than 1 in 500 in most of the population. The asbestos fibres counted were amosite (75%), crocidolite (18%), other amphiboles (5%) and chrysotile (2%).

**Conclusions:**

The approximate linearity of the dose–response together with lung burden measurements in younger people will provide reasonably reliable predictions of future mesothelioma rates in those born since 1965 whose risks cannot yet be seen in national rates. Burdens in those born more recently will indicate the continuing occupational and environmental hazards under current asbestos control regulations. Our results confirm the major contribution of amosite to UK mesothelioma incidence and the substantial contribution of non-occupational exposure, particularly in women.

**What this paper adds**

- Britons born before the 1960s have the highest mesothelioma death-rate worldwide, reflecting high occupational asbestos exposure in men and widespread environmental exposure in both sexes before 1980, when asbestos use virtually ceased in Britain.
- The risk to younger people from asbestos still present in many buildings is not known but could be substantial.
- We have shown that lifetime mesothelioma risk is approximately 0.020% per 1000 asbestos fibres per gram of dry lung tissue over a more than 100-fold range, from 1 to 4 in the most heavily exposed building workers to less than 1 in 500 in most of the population.
- This will enable the risk from current asbestos exposure to be estimated in people born since the 1970s for whom lung samples are available (eg, resected lung cancer or pneumothorax patients), both in occupations at potential risk such as builders and teachers and in the general population.
- Such data will provide a rational basis for regulations on worker protection and asbestos monitoring and abatement, and for predicting UK mesothelioma rates over the next 50 years.

## Background and aims

**Figure 1:**
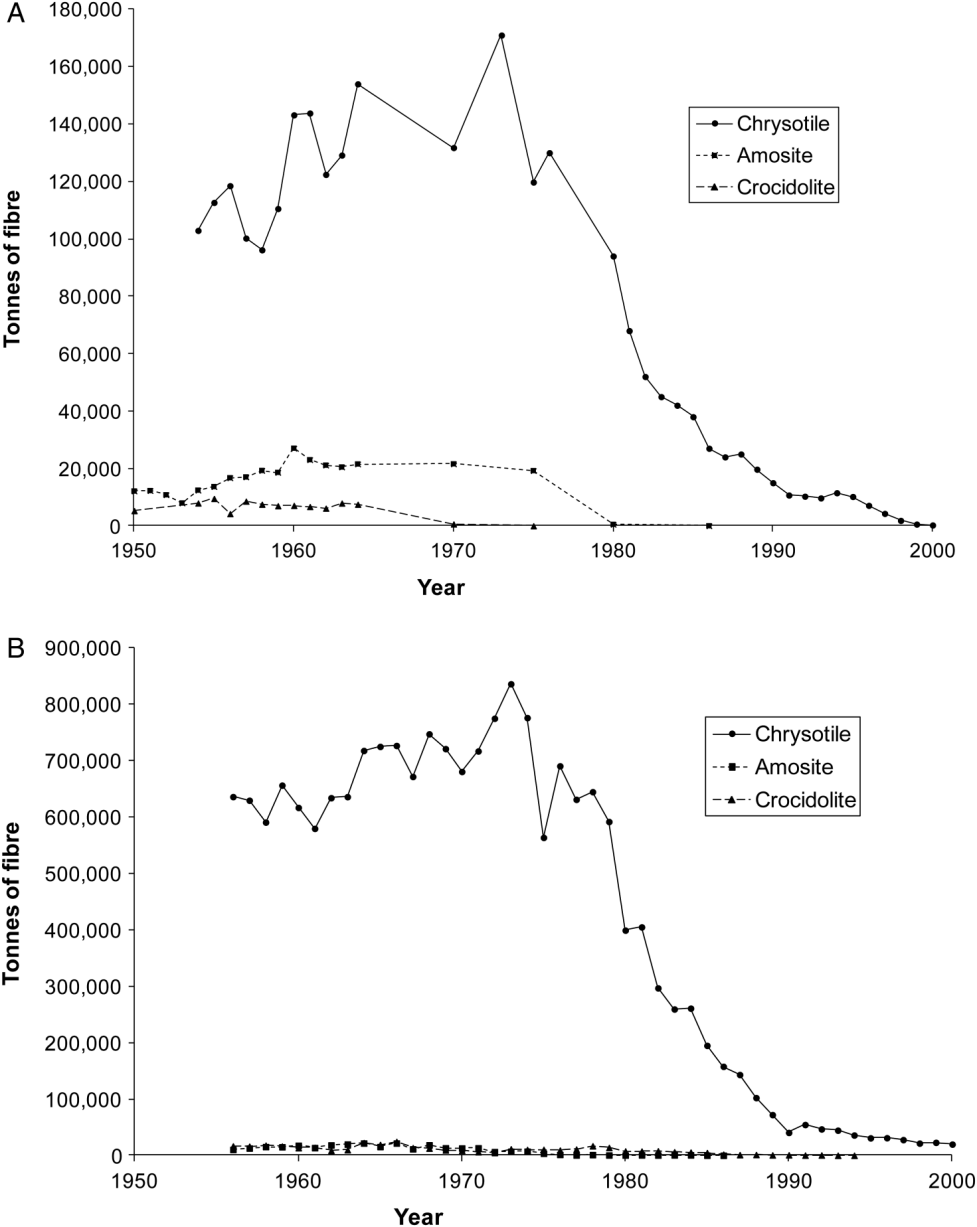
(A) UK Asbestos imports from 1950 to 2000.^27–30^ (B) US Asbestos imports from 1956 to 2000.^30^

A large amount of asbestos remains in many older buildings and there is continuing concern about environmental exposure to occupants and occupational exposure during maintenance, renovation and demolition in homes, schools and workplaces. The resulting mesothelioma risks cannot be calculated by extrapolation from historical occupational cohort studies because lifetime average airborne exposure levels in the breathing zone cannot be estimated even approximately either for the general public or for plumbers, electricians and other building or demolition workers. Asbestos lung burden is the only indicator of cumulative lifetime exposure that can be measured reliably in a population-based study. We have therefore developed a dose–response model in a population-based series of mesothelioma and resected lung cancer patients with occupational histories obtained by personal interview and measured lung burdens. This will enable future mesothelioma rates to be predicted from lung burdens in occupational groups and in the general population for people born after 1965 who began work after 1980 when asbestos use had virtually ceased in Britain (figure 1).

## Methods

### Source of samples

The methods and results of the MALCS case–control study have been described elsewhere.^1^ Telephone interviews on lifetime occupational^2^ history were conducted between 2001 and 2006 on 622 patients with mesothelioma and 1420 population controls. We also interviewed 420 patients with resected lung cancer born since 1940 for whom lung samples could be obtained as a control group for lung burden analyses. Patients with lung cancer and mesothelioma identified through chest physicians, lung cancer nurse specialists and Hospital Episode Statistics (HES) notifications were recruited from 170 hospitals throughout Britain.^1^ ^2^ Resected lung cancers provide the only adequate national source of lung samples in people who can be identified systematically, are available for interview and have an age distribution similar to mesothelioma. Only a small proportion of all lung cancers are caused by asbestos, so the asbestos lung burdens of this national sample are reasonably representative of the general population except for a few per cent with very high burdens. Written informed consent was obtained from 346 (77%) patients with mesothelioma and their next of kin for postmortem samples to be analysed and from 406 (96%) patients with lung cancer for analysis of resected tissue. Transmission electron microscopy (TEM) analysis was performed on samples as they became available, and 133 mesothelioma samples and 262 lung cancer samples were analysed. All were born since 1940 except 11 female mesotheliomas born 1925–1939. The study was approved by South Thames Multicentre Research Ethics Committee.

### Occupational classification

Job titles were assigned to Standard Occupational Classification 1990 (SOC 90) and Standard Industrial Classification 1992 (SIC 92) codes and grouped into main job categories. Proportional mortality ratios based on all mesothelioma deaths in Britain aged 16–74 years between 1991 and 2000^3^ provided the basis for this categorisation.^1^ ^2^ Subjects were assigned to the highest-ranking occupation they had worked in irrespective of duration. Previously reported ORs for these categories^1^ are shown in table 3.

### Lung sample preparation and TEM

Lung samples were anonymised and sent to the Health and Safety Laboratory (HSL) for TEM counting of asbestos fibres longer than 5 µm (appendix 2). The target analytical sensitivity, 0.01 mf/g (million fibres per dry gram), was achieved in all but 2.8% of the samples (2/133 mesotheliomas, 9/262 lung cancers). Sensitivity was increased to 0.003 mf/g for a subset of samples in which five or fewer asbestos fibres were originally counted.

### Statistical methods

**Figure 2:**
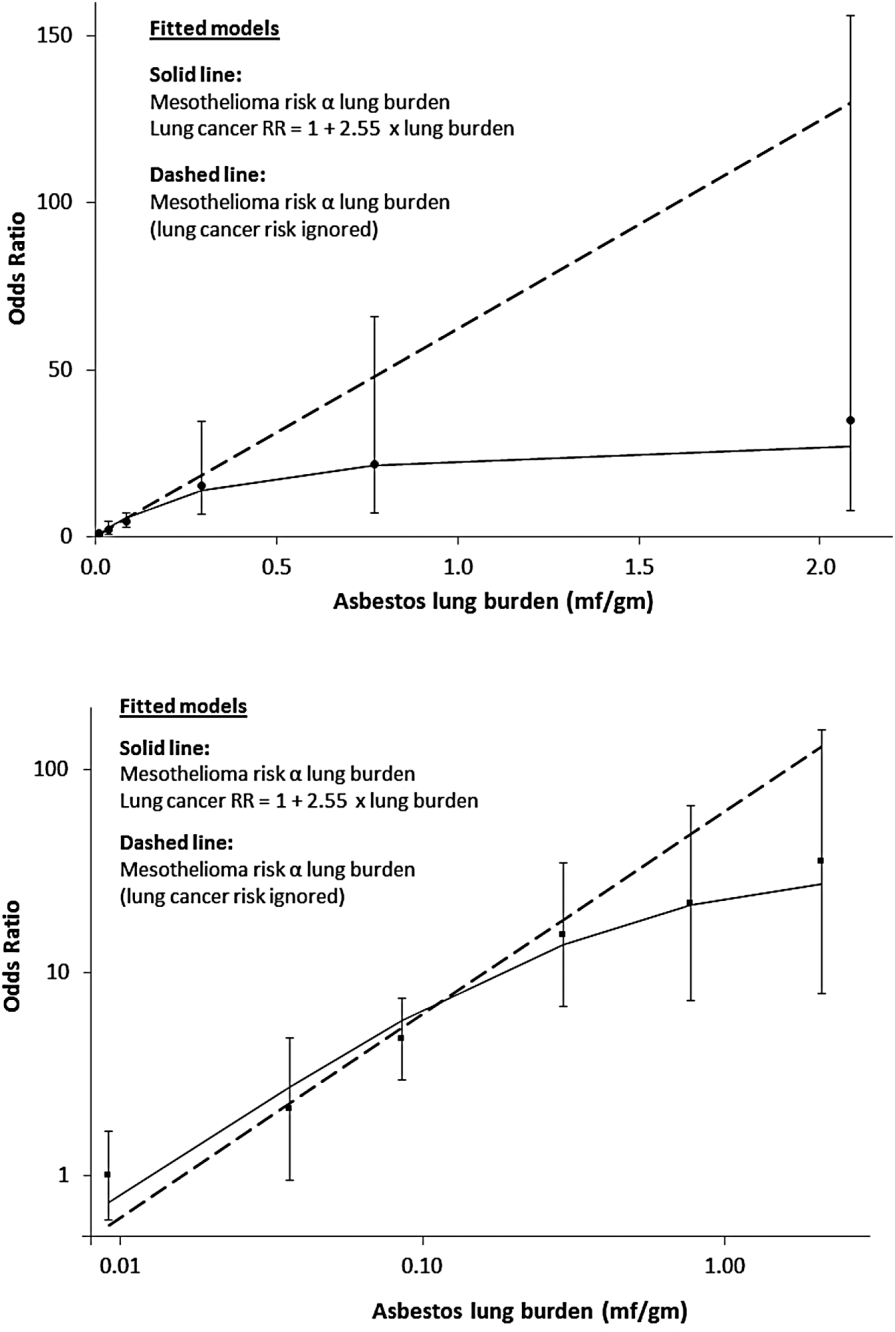
Mesothelioma ORs (95% floating CIs) in men using resected lung cancers as controls, and asbestos lung burden: upper graph linear axes, lower graph logarithmic axes. When the lung cancer risk caused by asbestos is ignored the fit of the linear model is significantly worse (p=0.02; dashed line).

The analyses are described in appendix 1. The fitted model estimates and adjusts for the effect of using lung cancers as controls. At low doses the mesothelioma:lung cancer OR will reflect the true mesothelioma dose–response, but as lung burden increases there is increasing downward curvature (solid line in figure 2) due to the increasing proportion of lung cancers caused by asbestos. This model was used to estimate the distribution of lung burdens in British men born in 1945, and hence to calculate their lifetime risks for mesothelioma and lung cancer as a function of asbestos lung burden (see table 2 footnotes). Our mesothelioma cases are well represented by this birth cohort, as their median date of birth was September 1944. The 1945 birth cohort's future age-specific death-rates were estimated by unadjusted age and birth cohort analysis of British male mesothelioma and lung cancer death-rates in 5-year age-groups (35–39 to 85–89) and periods (1990–1994 to 2005–2009). Our dose–response model is linear, so predicted mesothelioma and excess lung cancer age-specific death rates are both proportional to mean lung burden in each lung burden category. The lifetime risk (probability of dying by age 90) was calculated actuarially in each lung burden category assuming current (2013) UK rates for all other causes of death. These lifetime risks were standardised to the projected probabilities of dying by age 90 for mesothelioma (0.86%) and lung cancer (4.67%) of all British men born in 1945.

The main results are based on total asbestos fibre burden irrespective of fibre type. The mesothelioma risk per fibre of crocidolite relative to amosite was estimated by logistic regression, fitting the weighted sum of the amosite and crocidolite lung burdens, ignoring other fibre types (which constituted only 7% of counted fibres) and adjusting the crocidolite:amosite weighting to give the best-fitting model.

## Results

### Dose–response for mesothelioma and lung cancer

Table 1 shows the distribution of asbestos lung burdens in mesotheliomas and resected lung cancers. The estimated ORs for males and females combined (last row) are adjusted for period of birth (1940–1944, 1945–1949, 1950–1954 and 1955+) and sex, although neither was significant (p=0.6 for sex, p_trend_=0.5 for period of birth). There were too few women for separate analysis, and further model-fitting was confined to men. The reference group for the mesothelioma ORs in figure 2 and table 2 is the lowest lung burden category (<0.025 mf/g, average 0.0092 mf/g).

**Table 1:** Distribution of asbestos lung burdens (million fibres longer than 5 µm per dry gram) in men and women

	TEM asbestos lung burden in million fibres per dry gram (Average lung burden for lung cancers in brackets)*						
Source of sample	0− (0.0092)	0.025− (0.0364)	0.05− (0.0854)	0.2− (0.2930)	0.5− (0.7690)	≥1.0 (2.0829)	Total
Males							
Mesothelioma	18 (16.8%)	8 (7.5%)	33 (30.8%)	21 (19.6%)	15 (14.0%)	12 (11.2%)	107 (100%)
Lung cancer	105 (57.7%)	22 (12.1%)	41 (22.5%)	8 (4.4%)	4 (2.2%)	2 (1.1%)	182 (100%)
OR (95% CI)	1.00 (ref)	2.12 (0.82 to 5.49)	4.70 (2.38 to 9.25)	15.31 (5.89 to 39.8)	21.88 (6.52 to 73.4)	35.00 (7.22 to 169.6)	
Females							
Mesothelioma	13 (50.0%)	2 (7.7%)	7 (26.9%)	4 (15.4%)			26 (100%)
Lung cancer	62 (77.5%)	11 (13.8%)	6 (7.5%)	1 (1.3%)			80 (100%)
OR (95% CI)	1.00 (ref)	0.87 (0.17 to 4.39)	6.36 (1.89 to 21.44)	19.08 (1.97 to 184.91)			
Both sexes†							
Mesothelioma	26	8	38	23	15	12	122
Lung cancer	167	33	47	9	4	2	262
Adjusted OR (95% CI)	1.00 (ref)	1.51 (0.62 to 3.65)	4.81 (2.61,8.85)	13.91 (5.69 to 34.0)	21.52 (6.45 to 71.7)	30.70 (6.38 to 147.7)	

†Data for both sexes combined exclude 11 female mesothelioma cases born 1925–1939: 5 (TEM <0.025), 2 (TEM 0.025-), 2 (TEM 0.05-), 2 (TEM 0.2-). ORs for both sexes combined are adjusted for sex and year of birth (1940–1944, 1945–1949, 1950–1954).

*Mean lung burden of lung cancer samples in each category except the highest (≥1 mf/g). One lung cancer with 22.0 mf/g was recoded as 2.08 mf/g, the mean for the other lung cancer and the 12 mesotheliomas ≥1 mf/g. The mean for samples ≥1 mf/g was also set as 2.08 mf/g. Retaining the original value has little effect on the fitted model but distorts the lung burdens shown in table 3.

TEM, transmission electron microscopy.

**Table 2:** Observed distribution of asbestos lung burdens in male mesotheliomas and lung cancers, estimated distribution in the UK male population (central cohort born 1945), and predicted lifetime risks for mesothelioma and lung cancer

	TEM asbestos burden (million fibres ≥5 µm in length per dry gram)						
	0−	0.025−	0.05−	0.2−	0.5−	≥1.0	All men
Mean lung burden (mf/g)*	0.00918	0.0364	0.0854	0.293	0.769	2.08	
	*Distribution of lung burdens in mesotheliomas and lung cancers (from table 1) and fitted OR model (solid line in figure 2)*						
Mesotheliomas (mean lung burden 0.430 mf/g)	16.8%	7.5%	30.8%	19.6%	14.0%	11.2%	100%
Lung cancers (mean lung burden 0.082 mf/g)	57.7%	12.1%	22.5%	4.4%	2.2%	1.1%	100%
Mesothelioma/lung cancer OR							
Observed	1.0 (ref)	2.12	4.70	15.31	21.88	35.00	
Fitted†	0.74	2.74	5.76	13.79	21.35	27.13	
	*Estimated distribution of lung burdens and resulting mesothelioma and lung cancer risks due to asbestos in the UK male population born in 1945*						
Lifetime mesothelioma risk‡	0.18%	0.72%	1.66%	5.45%	12.91%	26.99%	0.86%
Mesothelioma SMR§	21	83	193	633	1501	3137	100
Lifetime lung cancer risk‡	4.55%	4.83%	5.34%	7.41%	11.67%	20.64%	4.67%
Lifetime excess lung cancer risk‡	0.10%	0.38%	0.89%	2.97%	7.22%	16.20%	0.47%
Lung cancer SMR¶	97	103	114	159	250	442	100
UK population (estimated mean lung burden 0.047 mf/g)**	63.08%	12.38%	20.70%	2.82%	0.83%	0.19%	100%

*Mean lung burden of lung cancer samples in each category except the highest (≥1 mf/g). One lung cancer with 22.0 mf/g was recoded as 2.08 mf/g, the mean for the other lung cancer and the 12 mesotheliomas ≥1 mf/g. The mean for samples ≥1 mf/g was also set as 2.08 mf/g. Retaining the original value has little effect on the fitted model but distorts the lung burdens shown in table 3.

†Solid line in figure 2.

‡Actuarial calculation of probability of dying by age 90 from projected mesothelioma and lung cancer rates assuming national rates for other causes of death.

§Proportional to mean lung burden.

¶Proportional to 1+2.55× (mean lung burden).

**Proportional to number of lung cancers divided by lung cancer SMR.

TEM, transmission electron microscopy, SMR, Standardised Mortality Ratio.

In the fitted model risks for both mesothelioma and excess lung cancer are proportional to lung burden. The estimated coefficients from the fitted model (solid line in figure 2) are 82.2 (95% CI 54.3 to 124.5) per mf/g for the OR for mesothelioma and 2.55 (95% CI 0.62 to 10.37) per mf/g for the increase in the lung cancer RR. The corresponding projected lifetime risks and SMRs in each lung burden category are shown in table 2 for the cohort of British men whose central date of birth is the beginning of 1945. (The median date of birth of our mesothelioma cases was September 1944.) The predicted lifetime excess risk for lung cancer due to asbestos (0.47%) is 55% of that for mesothelioma (0.86%). Mesothelioma and excess lung cancer risks in each category and overall are proportional to mean lung burden under this linear model, which implies a lifetime mesothelioma risk of 0.020% per 1000 asbestos fibres/g. The proportion of men with lung burdens exceeding 1 mf/g is 11.2% (12/107) in mesotheliomas, 1.1% (2/182) in lung cancers and is estimated as 0.19% in the UK male population. The estimated mean lung burden for the 1945 male birth cohort is 0.047 mf/g.

### Occupation and lung burden

**Figure 3:**
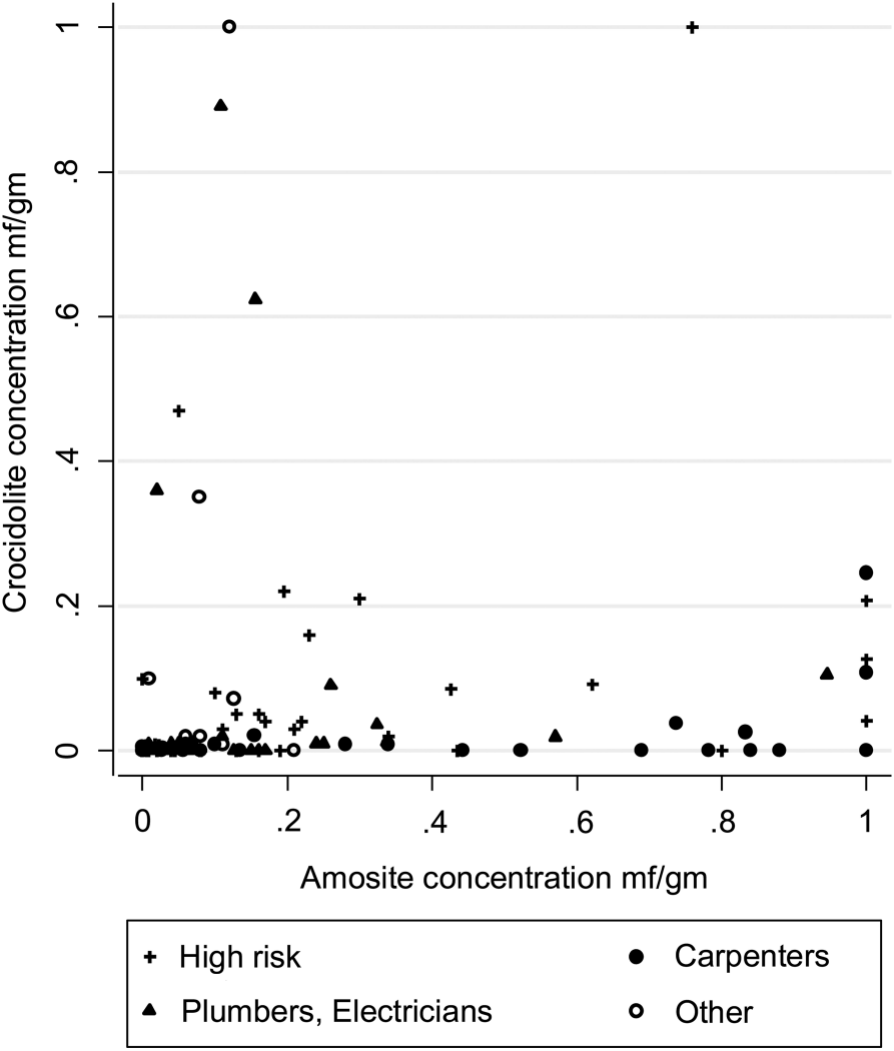
Amosite and crocidolite lung burdens and occupational categories in male mesotheliomas. (Burdens >1 million fibres/g truncated to 1 mf/g).

Amosite and crocidolite lung burdens among male mesotheliomas are shown in figure 3 by occupational category as previously defined^1^ (highest lifetime category irrespective of duration). Concentrations are generally higher for amosite than crocidolite. The highest amosite levels are predominantly in carpenters, while four of the five men with the highest crocidolite levels reported exposure to sprayed crocidolite. Table 3A, B show TEM results for males and females respectively by occupational category. Mesothelioma ORs (from Rake *et al*^1^) and mean lung burdens for each type of asbestos are also shown. Mean lung burdens are higher for mesothelioma than for lung cancer within each occupational category and increase with increasing occupational OR. Only six (3.3%) of 182 lung cancers in men and none of the mesotheliomas or lung cancers in women had lung burdens above 0.5 mf/g. In contrast, 27 (25.2%) of the male mesotheliomas were above 0.5 mf/g. All 27 had a high-risk occupational history and 16 had worked as a carpenter, plumber, electrician or decorator. Construction and medium risk industrial workers with lung cancer had much lower lung burdens, with 50 (61.7%) below 0.025 mf/g and only three (3.7%) above 0.2 mf/g. No asbestos fibres were detected in 4 of the 14 male mesotheliomas with lung burdens <0.025 mf/g who worked in high risk or construction jobs. These four men all reported short or occasional asbestos exposure in their work. Levels are much lower in women, with the highest concentrations in those who reported domestic exposure (table 3B).

**Table 3:** TEM asbestos lung burdens (million asbestos fibres ≥5 µm in length per dry gram) by most hazardous occupation

Panel A: Males born since 1940													
Highest occupational exposure category	Asbestos lung burden (million fibres per dry gram)							Meso OR vs population controls*	Mean asbestos lung burden (million fibres per dry gram)				
	0−	0.025−	0.05−	0.2−	0.5−	≥1.0	Total		Amosite	Crocidolite	Other amphiboles	Chrysotile	All asbestos
Non-construction high-risk occupations													
Mesothelioma	5	3	11	7	6	4	36	17.5	0.375	0.094	0.014	0.005	0.487
Lung cancer	15	1	9	1	3	0	29		0.100	0.013	0.003	0.005	0.121
Carpenters													
Mesothelioma	1	2	5	3	7	5	23	34.2	0.811	0.021	0.016	0.003	0.852
Lung cancer	3	1	3	2	0	0	9		0.088	0.002	0.005	0.000	0.095
Plumbers, electricians and painter/decorators													
Mesothelioma	5	3	10	8	2	2	30	15.9	0.148	0.074	0.004	0.002	0.228
Lung cancer	12	4	5	3	1	1	26		0.095	0.040	0.006	0.001	0.143
Other construction or other reported exposure													
Mesothelioma	3	0	2	0	0	1	6	5.1	0.056	0.192	0.000	0.000	0.248
Lung cancer	28	3	13	1			45		0.027	0.004	0.002	0.002	0.036
Medium risk industrial													
Mesothelioma	3	0	2	3	0		8	4.1	0.069	0.057	0.010	0.001	0.137
Lung cancer	22	5	7	1	0	1†	36		0.078†	0.015†	0.005	0.001	0.098†
Domestic exposure													
Mesothelioma	0	0	2				2	2.1	0.035	0.060	0.000	0.000	0.094
Lung cancer	4	4	0				8		0.009	0.004	0.006	0.001	0.020
Low-risk occupations													
Mesothelioma	1	0	1				2	1.0 (ref)	0.015	0.018	0.005	0.000	0.038
Lung cancer	21	4	4				29		0.010	0.003	0.007	0.002	0.021
Total													
Mesothelioma	18	8	33	21	15	12	107		0.351	0.073	0.010	0.003	0.438
Lung cancer	105	22	41	8	4	2	182		0.058†	0.012†	0.004	0.002	0.077†
High−risk occupations													
Mesothelioma	1 (1)	1 (1)	0	0			2 (2)	4.8	0.025	0.000	0.000	0.000	0.025
Lung cancer	3	1	0	0			4		0.010	0.000	0.000	0.003	0.013
Medium risk industrial													
Mesothelioma	5 (1)	1 (1)	1	0			7 (2)	2.4	0.004	0.027	0.003	0.000	0.034
Lung cancer	20	1	4	0			25		0.012	0.002	0.003	0.002	0.019
Domestic exposure													
Mesothelioma	2 (2)	0	2 (1)	3 (1)			7 (4)	1.9	0.103	0.077	0.004	0.003	0.186
Lung cancer	13	6	0	1			20		0.018	0.006	0.002	0.001	0.027
Low-risk occupations													
Mesothelioma	5 (1)	0	4 (1)	1 (1)			10 (3)	1.0 (ref)	0.067	0.017	0.001	0.003	0.087
Lung cancer	26	3	2	0			31		0.009	0.002	0.002	0.001	0.013
Total													
Mesothelioma	13 (5)	2 (2)	7 (2)	4 (2)			26 (11)		0.056	0.034	0.002	0.002	0.095
Lung cancer	62	11	6	1			80		0.012	0.003	0.003	0.001	0.019

*Male mesothelioma ORs from the original case–control study.^1^

†One lung cancer with 22.0 mf/g was recoded as 2.08 mf/g (see table 2 footnote *).

Retaining the original value increases the mean fibre count in lung cancers to 0.555 for amosite, 0.091 for crocidolite and 0.651 for all asbestos in the medium risk group, and to 0.153 for amosite, 0.027 for crocidolite and 0.186 for all asbestos in all male lung cancers.

TEM, transmission electron microscopy.

*Female mesothelioma ORs from the original case–control study.^1^

### Effects of fibre type and size

Figure 1 shows UK asbestos imports since 1954. Of the five million tonnes imported over this period 89% was chrysotile, 9% amosite and 2% crocidolite. Crocidolite use had ended by 1970 and amosite by 1980. Chrysotile imports had fallen by 90% by 1990 and ended by 2000. Few of the asbestos fibres detected were chrysotile, which disappears from the lung with a half-life of a few months.^4^ The majority of counted asbestos fibres were amosite (75%) or crocidolite (18%), with much lower numbers for chrysotile (1.9%), tremolite (1%), anthophyllite (2%), actinolite (0.6%) and uncharacterised amphiboles (1.7%). Burdens of chrysotile and these other amphiboles were correlated with the total fibre burden and were too low for their effect on risk to be estimated. Table 4 (see online supplementary material) shows mesotheliomas and lung cancers classified by amosite and crocidolite concentration, ignoring other fibres. A logistic model in which one crocidolite fibre is equivalent to 1.3 (95% CI 0.4 to 3.3) amosite fibres gave the best fit.

Distributions of fibre length were similar irrespective of disease status, fibre type or lung burden, the overall distribution being 76.1% 5–10 µm, 21.2% 10–20 µm and 4.5% >20 µm. Median widths were 0.09 µm (chrysotile), 0.17 µm (crocidolite), 0.30 µm (amosite), 0.49 µm (tremolite), 0.58 µm (anthophyllite) and 0.61 µm (actinolite). No significant association was seen between disease status and fibre dimension after stratifying by fibre type.

## Discussion

### Dose–response

This is the first study with occupational histories obtained by personal interview and asbestos lung burdens measured by TEM from a large population-based series of patients with mesothelioma. Our fitted model estimates and adjusts for the effect of using lung cancers as controls. Table 2 shows that the effect is small among the 96% of men whose lung burdens are less than 0.2 mf/g (lung cancer SMR <1.1) but implies that the majority of lung cancers are caused by asbestos in the 1% of men whose lung burdens are above 0.5 mf/g. As expected, mean lung burdens are consistently higher for mesothelioma than for lung cancer within each occupational category and increase with increasing occupational OR. Our results confirm that most mesotheliomas are caused by asbestos even in those who never worked in asbestos-related occupations. Larger numbers and more sensitive TEM would be required to estimate the incidence of spontaneous mesotheliomas unrelated to asbestos, which is ignored in our modelling. The ORs in figure 2 are scaled to make the observed OR unity in the lowest exposure group (<0.025 mf/g). The fitted value (solid line in figure 2) equals 0.74 in this group. This small and non-significant difference corresponds to a lifetime spontaneous mesothelioma risk of about 1 per 2000, almost an order of magnitude greater than early estimates of the spontaneous incidence in both sexes.^5^

Our estimates of lifetime excess risk due to asbestos in British men born in 1945 are 0.86% for mesothelioma and 0.47% for lung cancer, a ratio of excess lung cancer to mesothelioma of 0.55. Two independent sources also suggest that asbestos causes more mesotheliomas than lung cancers in British men. An analysis of proportional mortality ratios for different occupational groups concluded that the ratio of excess lung cancer to mesothelioma in British men is about 0.7.^6^ The ratio was 1.3 (1795 deaths, 965 expected for lung cancer and 639 mesothelioma deaths) in men in the Great Britain Asbestos Workers prospective study, but adjustment for smoking reduced the estimate for the general population to 0.7.^7^ The ratio would be substantially higher in the earlier birth cohorts included in that study due to their higher smoking-related lung cancer rates, so our estimate of the ratio for the 1945 birth cohort (0.55) may be approximately correct despite the imprecision of the estimated increase in lung cancer RR (2.55 per mf/g, 95% CI 0.62 to 10.37). The lifetime lung cancer risk at a given asbestos lung burden will continue to fall in later generations because they smoke less but the lifetime mesothelioma risk will be higher because they will live longer. The model used by Tan *et al*^8^ implies that mesothelioma incidence in the 1945 male birth cohort will increase less steeply with age than in earlier generations. That analysis (updated to 2013) predicts that their lifetime mesothelioma risk will be 0.72%. If this proves more accurate than our simple projection (0.86%) the mesothelioma risk per 1000 fibre/g should be reduced from 0.020% to 0.017%. The lifetime risk per 1000 f/g in women will be slightly greater due to their longer life expectancy.

Linear dose–response is the most important assumption underlying the risk estimates for mesothelioma in table 2, which are constrained to match the predicted lifetime risk for the UK male population born in 1945. The relationship between mesothelioma risk and lung burden of asbestos measured by TEM is perhaps the only example of a major human carcinogen for which the data span such a wide range of measured dose and risk. Stomata in the parietal pleura where long fibres congregate may be the main site of carcinogenesis,^9^ but for fibres of specified dimension it seems reasonable to assume a linear relationship between inhaled dose, fibre concentration in pleural stomata and our measurements in lung parenchyma. Linear dose–response might therefore be expected if mesothelioma were initiated by a single asbestos fibre in a single cell, but tumour progression may also involve dose-related local inflammatory processes.^9^ Doll and Peto^10^ observed a quadratic dose–response for cigarette smoking and lung cancer indicating both early and late effects in lung carcinogenesis and suggested that the linear relationship seen in other studies reflected inaccurate measurement of lifelong smoking rates. Our lifetime mesothelioma risk estimate of 0.020% per 1000 asbestos fibres/g provides a reasonably reliable basis for predicting future mesothelioma rates in birth cohorts born since 1965 from their average asbestos lung burdens. For estimating the exposure level or lung burden that would cause lifetime mesothelioma risks of the order of 1 in 100 000, an order of magnitude less than the estimated spontaneous rate^5^ and two orders of magnitude below the range in our data, risk assessment conventions rather than epidemiology must determine the basis of the extrapolation. We did not include any peritoneal mesotheliomas, which are both under- and over-diagnosed due to confusion with cancers of the ovary and other abdominal sites.^11^ Peritoneal mesotheliomas constitute less than 4% of all mesotheliomas in the UK, and if the dose-response with amphibole exposure is quadratic^13^ the proportion will be even less at lower dose levels.

### Effects of amphiboles and chrysotile

Asbestos consumption per head since the 1950s was similar in Britain and the US for chrysotile and crocidolite but amosite consumption was about five times higher in Britain (figure 1). This seems likely to explain the fivefold greater mesothelioma death-rate in Britain than in the USA among men born around 1945.^1^ UK crocidolite imports ceased by 1970, a decade earlier than amosite imports, but some exposure continued. The median concentration of crocidolite fibres was 0.009 mf/g in male mesothelioma cases born 1940–1949, and was still 0.004 mf/g in those born after 1950 who started work around or after the time that crocidolite use ended. Our model suggests that the mesothelioma risk per fibre is approximately 1.3 (95% CI 0.4 to 3.3) times higher for crocidolite than for amosite. The proportion of TEM fibres with width >0.2 µm and therefore observable by phase contrast optical microscopy (PCOM) was 38% for crocidolite and 75% for amosite. Our estimate of the risk per fibre of crocidolite relative to amosite is thus approximately doubled to give 2.6 (95% CI 0.8 to 6.6) for PCOM data, statistically consistent with the estimate of five based on cohort studies with PCOM fibre counting of air samples.^12^

Other evidence shows that chrysotile causes a much lower mesothelioma risk than amosite or crocidolite.^12^ ^13^ The rapid clearance of chrysotile from the lung with a half-life of a few months^3^ explains its virtual absence in our samples, and implies that we cannot estimate its effects except by noting that amphibole lung burdens account very well for mesothelioma incidence. Rasmuson *et al*^14^ reported a good correlation between lung burden and estimated cumulative exposure for amphiboles but not for chrysotile. The prolonged heavy chrysotile exposure that occurred in some British factories before the 1932 Asbestos Industry Regulations were introduced caused an Standardised Mortality Ratio (SMR) of more than 10 for lung cancer in chrysotile textile workers,^15^ but the contribution of chrysotile to current UK lung cancer rates is not known and may be impossible to ascertain.

### Biopersistence of amphiboles

Earlier studies showing an approximately linear relationship between amphibole lung burden and mesothelioma risk reported higher lung burdens in cases and controls^16–18^ than we observed. The half-life of amphiboles in the lung has been estimated as about 6–10 years^4^ ^19–21^ for crocidolite and perhaps 20 years for amosite.^4^ Comparison of the distributions of lung burdens in this study and in an earlier study of 69 British men who died of mesothelioma^17^ also suggests a longer half-life for amosite. These men, like our cases, were all born since 1940, and most died in 1994–1995, about 9 years before our cases. The respective proportions of male mesotheliomas exceeding 0.1 mf/g in our data and in the earlier series were 42.1% and 46.4% for amosite and 11.2% and 29.0% for crocidolite (fibres >6 µm; JC McDonald and B Armstrong, personal communication). However, we have not attempted to adjust our data for elimination, for two reasons. First, these men were born since 1940 and were exposed predominantly between 1960 and the late 1970s when amosite exposure ended, so their average intervals from exposure to lung sampling were similar. Second, our lung samples were obtained more than 20 years after substantial amphibole exposure had ceased. If as Tossavainen *et al*^22^ suggest, further clearance of long amphibole fibres will be minimal, further studies over the next decade should show a similar dose–response. A higher proportion of inhaled fibres from more recent environmental exposure will be retained, however, somewhat exaggerating mesothelioma risks predicted from amphibole lung burdens in those born more recently.

Conversely, a residence time model in which earlier exposure causes a higher lifetime risk and later exposure is discounted^8^ would imply higher dose-specific risks in younger people, particularly for environmental exposure which presumably began in childhood.

### Environmental asbestos exposure

Our case–control analysis suggested that 14% of male and 62% of female mesotheliomas were not attributable to occupational or domestic asbestos exposure.^1^ Among men and women with only low-risk occupations 6 of 12 mesotheliomas and 6 of the 60 lung cancers had lung burdens exceeding 0.05 mf/g (table 3). Three of these six mesotheliomas and one of the six lung cancers mentioned potential asbestos exposure at work (one occasionally handling sealed asbestos waste, one using asbestos ironing boards at work and two office workers in companies handling building materials). These potential exposures, which had not been classified as substantial in coding these occupational histories, suggest that approximately 25% (3/12) of mesotheliomas in apparently low-risk occupations may be due to such work-related exposures.

## Conclusion

Our results confirm the major contribution of amosite to UK mesothelioma incidence and the substantial contribution of non-occupational asbestos exposure, particularly in women.^1^ The overall distribution of asbestos lung burdens in British men born in the 1940s and their resulting mesothelioma and lung cancer risks are summarised in table 2. The lowest exposure category (<0.025 mf/g) includes 17% of mesotheliomas and 63% of the population, while 45% of mesotheliomas but only 4% of the population are above 0.2 mf/g. The approximate linearity of the dose–response together with lung burden measurements in younger people will provide reasonably reliable predictions of future mesothelioma rates in those born since 1965 whose risks cannot yet be seen in national rates. Burdens in those born more recently will indicate the continuing occupational and environmental hazards under current asbestos control regulations. Similar population-based studies with uniform TEM fibre counting methods in other countries, together with the international mesothelioma death-rates now available following the introduction of ICD-10 with a separate cause of death code for mesothelioma, would provide a worldwide perspective on future mesothelioma rates and more precise estimates of the risk per fibre for different amphiboles. Local studies are needed to estimate the risk per fibre, lung burdens in younger people, and hence the continuing environmental hazard from amphibole contamination in areas such as Libby, Montana^23^ and from other naturally occurring asbestiform fibres such as erionite in Turkey and elsewhere.^24^ The extent to which mesotheliomas in chrysotile workers are due to tremolite contamination or previous amphibole exposure could also be tested.^25^ If most mesotheliomas are due to amphibole exposures, the quantitative relationship we have observed between amphibole lung burden and mesothelioma risk should also be seen in the USA and in Eastern European and South American countries where amphiboles are reported to have constituted a much lower proportion of asbestos consumption than in the UK.

## Supplementary Material

Web table

## Appendix 1: Statistical methods

### Linear dose–response model

In the ith lung burden category there are l(i) lung cancers with mean asbestos lung burden d(i) and m(i) mesotheliomas. If mesothelioma risk and excess lung cancer relative risk both increase linearly with increasing lung burden, with slope b for mesothelioma and k for lung cancer, the expected ratio of mesothelioma to lung cancer is proportional to [b·d(i)]/[1+k·d(i)].

The slopes b and k are estimated by maximum likelihood from the log(odds), which are treated as independent with normally distributed error variances v(i)=1/m(i)+1/l(i). Thus

1

The constant determines the scale of mesothelioma risk (odds, OR, lifetime risk or SMR). The constant was set as the observed value of log[m(1)/l(1)], the log(odds) in the lowest (reference) exposure category, giving the solid line in figure 2. ORs for each lung burden category, including the reference group, are shown in figure 2 with ‘floating absolute risk’ CIs corresponding to the log(odds) variances v(i).^25^ Taking group 1 as the reference group, the usual definition of the OR in group i is (true odds in group i)/(true odds in groups 1). The definition of the floating OR is (true odds in group i)/(observed odds in group 1). The denominator, the observed odds in group 1, is a known constant with zero variance so the error variance of log(floating OR) in category i equals v(i), the variance of log(odds).

### Distribution of lung burdens in the general population and corresponding lifetime risks

In the ith lung burden category the number of lung cancers that were not caused by asbestos is l(i)/(1+k·d(i)). We assume that the general population have the same distribution of lung burdens as these non-asbestos lung cancers, so the proportion p(i) in the ith lung burden category among British men in the same birth cohort (ie, born around 1945) is estimated in table 2 as:

The mean lung burden of men in this birth cohort is thus ∑p(i)·d(i). Their projected death-rates at each age for mesothelioma and lung cancer were calculated by unadjusted age and birth cohort analysis of British male mesothelioma and lung cancer death-rates in 5-year age-groups (35–39 to 85–89) and periods (1990–1994 to 2005–2009). For men with average lung burden d(i) these projected rates were multiplied by M·d(i) for mesothelioma and L·(1+k·d(i)) for lung cancer. Their lifetime risks (respective probabilities of dying by age 90 of mesothelioma and lung cancer) were then calculated actuarially, assuming current (2013) rates for all other causes of death. The constants M and L were adjusted to make the population averages of these lifetime risks equal the overall population projections for lung cancer (4.67%) and mesothelioma (0.86%).

### Comparison of amosite and crocidolite

The effect of crocidolite relative to amosite was estimated in a logistic model fitting log(lung burden of amosite plus crocidolite) as a continuous variable in which crocidolite fibres were given a weighting w. We fixed the offset using the estimated lung burden coefficient k from the unweighted model to give nested models and a likelihood-based CI for w. For the jth individual with amosite lung burden a_j_ and crocidolite lung burden c_j_

where offset=log[m(1)/l(1)]−log(1+k·d_j_).

### Appendix 2: Lung sample preparation and transmission electron microscopy

All lung tissue samples were sent to a pathology laboratory in Leeds for an initial assessment of their suitability. Thin tissue sections were microtomed from the waxed blocks for further assessment, before the blocks were de-waxed in xylene and washed in ether and microdissected to remove cancerous and fibrotic tissue. The samples were then anonymised and sent to the Health and Safety Laboratory (HSL) for quantitative transmission electron microscopy (TEM) analysis. At HSL a representative sample was taken from the tissue supplied and diced into cubes (approximately 3 mm sides) and dried overnight in a vacuum dessicator and then weighed to obtain the dry weight. The tissue was then digested in bleach and aliquots filtered onto membrane filters. The filter with the largest aliquot was ashed overnight under controlled conditions in a low temperature asher to further remove organic material. The ashed residual was resuspended in water and a range of aliquots filtered onto 0.2 µm pore size polycarbonate filters. When dry, strips of the final filters were carbon coated and sections cut out and transferred onto 200 mesh nickel index grids. Several grids from each filter were prepared by dissolving away the polycarbonate on a filter paper soaked with a mixture of 20% 1,2-diaminoethane and 80% 1-methyl-2 -pyrrolidone. This left a thin carbon film with the entrapped particles supported on the grid. New disposable containers and filtration equipment were used for each sample to avoid any cross-contamination and a process blank was run with each batch of analyses.

The prepared TEM grids were analysed in a FEI CM12 TEM equipped with an EDAX Inc beryllium window energy dispersive X-ray detector. Grid openings were step scanned on the fluorescent screen at 11k magnification to identify fibres (particles with parallel sides and >3:1 aspect ratio) over 5 µm in length. The length, width, type of diffraction pattern and quantitative weight percentage oxide composition from the energy dispersive X-ray analysis were recorded for each asbestos fibre found. Counting continued until at least 30 asbestos fibres had been identified, or until 0.1 mg of defatted dry lung had been analysed, giving an analytical sensitivity of 0.01 mf/g (million fibres per dry gram). This sensitivity was not achieved in 2.8% of the samples (2/133 mesotheliomas, 9/262 lung cancers), due to samples with low fibre concentrations but high amounts of other inorganic particles that required lower filter loadings and hence large areas of the filter to be analysed by TEM. The sensitivity was increased threefold to 0.003 mf/g by later extending the TEM analysis on newer equipment for a selected subgroup of samples which included 25 of 26 male and 7 of 8 female samples from patients with mesothelioma born in 1940 or later in which 5 or fewer asbestos fibres were originally counted.
