# Supplementary material for: Pleural mesothelioma and lung cancer risks in relation to occupational history and asbestos lung burden
Source: Occup Environ Med. 2015 Dec 29;73(5):290–9. doi: 10.1136/oemed-2015-103074 (PMC4853597; doi:10.1136/oemed-2015-103074)
Supplement: Web table [file oemed-2015-103074-s1.pdf]

Supplementary Table 4. Amosite and crocidolite TEM asbestos lung burdens in male and female mesothelioma and lung cancer patients and male odds ratios. The 12 mesotheliomas in women born 1925-39 are included and are also shown in brackets.

		Crocidolite lung burden (million fibres/gm)													
Amosite lung burden (million fibres/gm)		0-0.024		0.025-0.049		0.05-0.19		0.2-0.49		0.5-0.99		≥1.0		Total	
		M	F	M	F	M	F	M	F	M	F	M	F	M	F
0-0.024	Mesothelioma	21	14 (6)	0	0	2	2	1						24	16(6)
	Lung cancer	125	67	1	1	2	0	0						128	68
	**Male OR**	**1.0 (ref)**		**0.0**		**6.0**		∞						**1.1**	
0.025-0.049	Mesothelioma	9	2 (2)	0		0			1	0				9	3(2)
	Lung cancer	14	6	2		1			0	1				18	6
	**Male OR**	**3.8**		**0.0**		**0.0**				**0.0**				**3.0**	
0.05-0.19	Mesothelioma	26	3 (2)	2		4	2	3		2		1		38	5(2)
	Lung cancer	22	5	1		0	1	0		0		0		23	6
	**Male OR**	**7.0**		**11.9**		∞		∞		∞		∞		**9.8**	
0.2-0.49	Mesothelioma	9	2 (1)	3		3	1 (1)	1						16	3(2)
	Lung cancer	6	0	2		0	0	0						8	0
	**Male OR**	**8.9**		**8.9**		∞		∞						**11.9**	
0.5-0.99	Mesothelioma	7		2		2						1		12	0
	Lung cancer	1		0		2						0		3	0
	**Male OR**	**41.7**				**6.0**						∞		**23.8**	
≥1.0	Mesothelioma	3		1		2		2				0		8	0
	Lung cancer	0		0		1		0				1		2	0
	**Male OR**	∞		∞		**11.9**		∞				**0.0**		**23.8**	
Total	Mesothelioma	75	21(11)	8	0	13	5 (1)	7	1	2	0	2	0	107	27 (12)
	Lung cancer	168	78	6	1	6	1	0	0	1	0	1	0	182	80
	**Male OR**	**2.7**		**7.9**		**12.9**		∞		**11.9**		**11.9**			
